# Rotor-Stator Emulsification in the Turbulent Inertial Regime: Experiments toward a Robust Correlation for the Droplet Size

**DOI:** 10.1021/acs.langmuir.3c02868

**Published:** 2023-12-06

**Authors:** Roberta Campardelli, Giulia De Negri Atanasio, Claudia Carotenuto, Raffaella Griffo, Essam Nabil Ahmed, Manuel Corrales-González, Jiasen Wei, Peter Enos Tuju, Andrea Mazzino, Jan Oscar Pralits

**Affiliations:** † Department of Civil, Chemical and Environmental Engineering, University of Genoa, Genoa 16145, Italy; ‡ Department of Engineering, University of Campania “Luigi Vanvitelli”, Aversa, Caserta 81031, Italy

## Abstract

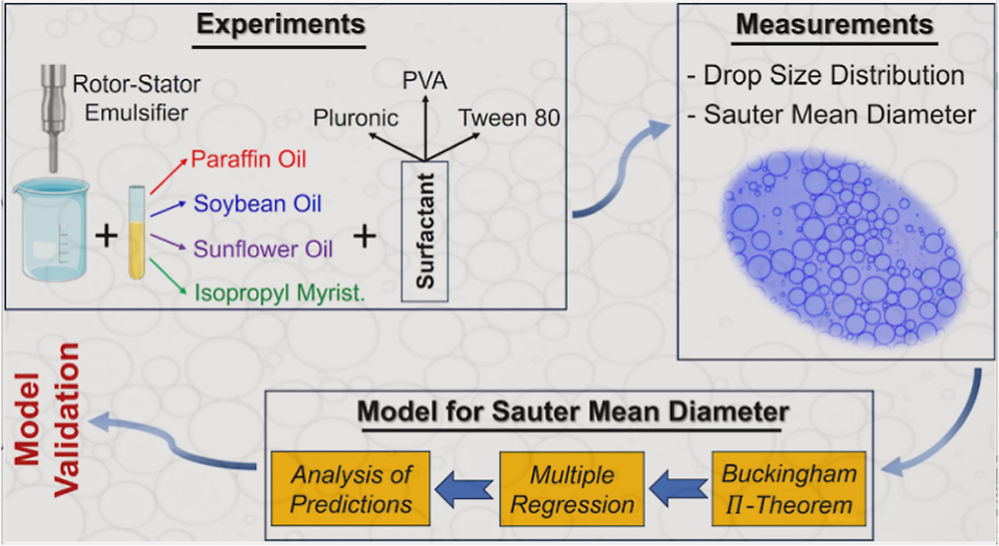

The Sauter mean diameter, *d*_32_, is a representative parameter in emulsions that indicates the average size of the oil droplets once the emulsion becomes stable. Several mathematical and physical approaches have been employed in the literature to seek expressions for *d*_32_ under different conditions. The present work sheds light on this rich literature and emphasizes that the characterization of emulsions is still a fertile field for investigation. In this paper, a new Π-theorem-based model to predict the normalized Sauter mean diameter for the specific case of rotor-stator emulsification is sought by applying a multiple regression analysis on experimental data of oil-in-water (O-W) emulsions produced using three different oils: paraffin, soybean oil, and isopropyl myristate, at different oil-to-water (O/W) ratios and rotor speeds. The proposed model quantifies the roles of the viscous, inertial, and interfacial tension forces, besides the O/W ratio, in the emulsification process within the turbulent inertial subrange. The developed empirical correlation is then contrasted with relevant literature models for reliability assessment; predictions of the present explicit model are proven to be more accurate for the fluid properties and the experimental conditions under study.

## Introduction

An emulsion is a mixture that results from two immiscible fluids, one being dispersed in the other, in the presence of surface-active agents, instigating the stabilization of the dispersed phase after fragmentation.^1,2^ Emulsification is rarely a spontaneous process; indeed, the free energy of emulsification is positive and, hence, the production of an emulsion is a process that requires the input of energy. This energy usually comes from mechanical shear provided by various types of mixers. The final droplet size of the emulsion depends not only on the chemistry but also on the amount of energy applied.^3^ Emulsions have been prepared on an industrial scale by a variety of emulsification equipment based on a similar operating principle, agitation. Rotor-stator homogenization belongs to this category.^4^ It consists of a central rotor and a stator with either vertical or slanted slots around the wall of the homogenizer cell. As the rotor rotates, it generates a vacuum to draw the liquid in and then pushes it out of the assembly, thereby resulting in circulation.

The rotor-stator homogenizer has been used for emulsification processes in a variety of applications^4−6^ such as food, beverages, cosmetics, paints, and nutraceutical industries, where a wide range of emulsion-based products are found.^1,5,7,8^ Compared with stirred tanks, another type of mixing device, the main advantage of the rotor-stator is its ability to create very high energy dissipation rates as the kinetic energy generated by the rotor is dissipated in the small stator volume, and high shear rates are generated in the rotor-stator gap.^9^ However, the physical mechanisms associated with rotor-stator emulsification lack comprehensive investigation for efficient production and industrial scale-up. In detail, the science of emulsion has to take into account the complex relationship between the properties of the droplets within an emulsion (e.g., concentration, size, charge, physical state, interfacial characteristics, and interactions) and emulsion physicochemical properties (e.g., stability, optical properties, rheology, and molecular distribution).^5,10,11^

The mean emulsion droplet size, commonly indicated as the Sauter mean diameter (*d*_32_), and the drop size distribution (DSD) are important parameters used to characterize the product of the emulsification process.^6,12−15^ These physical characteristics are of fundamental importance for suiting the field of application and for their strong influence on the stability, rheology, and interfacial physical properties of the emulsion. Some theoretical models for the oil droplet size in an emulsion are listed in Table S1. Many literature models employ Kolmogorov’s theory, which is pertinent to the turbulent inertial regime; the drop size is greater than the Kolmogorov length scale (, with ν and ϵ the kinematic viscosity and the turbulent kinetic energy dissipation rate, respectively) but smaller than the integral length scale (*L*_T_ ≈ the diameter of the rotor head). As can be noticed, the equilibrium droplet size depends on many factors, for instance, the Weber number,^16−24^ O/W ratio,^18,19,22,25^ and the Reynolds number (based on rotor diameter and speed).^21,26,27^ Furthermore, in practical applications, surfactants are usually used to produce stable emulsions at ambient conditions of temperature and pressure. In the presence of surfactants, the interfacial tension is altered, affecting the droplet stretching and breakup dynamics.

The current work aims to build a more comprehensive, robust, and novel analytical model for accurate estimation of the Sauter mean droplet diameter *d*_32_ of the emulsion droplets produced by a rotor–stator mixer. In the first part of this work, O-W-based emulsions were produced with a rotor-stator mixer for three different oils (paraffin, soybean oil, and isopropyl myristate) under three different O/W ratios (20/80, 10/90, and 5/95) and three rotor speeds (3000, 5000, and 7000 rpm), and the surfactant used was Tween 80 at 1%. The *d*_32_ of each emulsion was measured. In the second part, based on the experimental dataset, a mathematical model for the *d*_32_ prediction was proposed employing the Π-theorem dimensional analysis. The model is then validated against experimental data of an O-W-based emulsion with sunflower oil and isopropyl myristate with different surfactants. Finally, a comparison between the accuracy of the present model and models available in the literature is presented to show that the proposed model gives improved estimations of the Sauter diameter.

## Methodology

### Experimental Setup

#### Experimental Apparatus

A rotor-stator homogenizer (Silverson L5T), see Figure S1, was used to produce the emulsion. The rotor diameter was *D* = 3.2 cm. The volume of the mixing tank was 250 mL, and the produced emulsion volume was 100 mL. The first set of tests was carried out to investigate the effect of the oil phase, the O/W ratio, and the rotor speed. Three different oils were used: isopropyl myristate, soybean oil, and paraffin, and three different O/W ratios (on a mass basis) were tested: 20/80, 10/90, and 5/95, which are indicated in terms of the oil mass concentration as ϕ_*m*_ = 0.2, 0.1, and 0.05, respectively. The corresponding values of the oil volume concentration, ϕ_*v*_, can be simply evaluated based on the water-to-oil density ratio, ρ_*c*_/ρ_*d*_, as follows
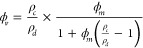
1

Further, three different rotor-stator speeds were investigated (3000, 5000, and 7000 rpm), and the surfactant employed was the polysorbate (Tween) 80 at a concentration of 1%, dissolved in the water phase. Briefly, the water phase was kept under agitation until the required rotational speeds were achieved. After one min, the oil phase, in the required concentration, was added slowly and left under emulsification at the fixed time of 6 min. The mixing time was chosen according to an optimization of the experiment duration.^28^

The main objective was to measure the Sauter mean diameter, defined as the diameter of a sphere that has the same volume/surface-area ratio as a particle of interest according to
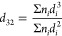
2where the index *i* indicates a size class, *n*_*i*_ is the number of drops in that particular class, and *d*_*i*_ is the corresponding nominal diameter.

#### Materials

Different emulsions were prepared using paraffin oil, commercial soybean oil, isopropyl myristate oil, and sunflower oil, purchased from Acros Organics (Geel, Belgium). The properties of the oils are listed in Table 1. The dynamic viscosities were gathered from the literature data.^29−37^ The densities of the oils were measured using a densimeter (Density Meter Anton Paar DM 4500 M) exploiting the pulsed excitation method. The interfacial tensions of oils in water/Tween 80 mixtures at 1% were measured using the tensiometer FTA1000 (First Ten Angstroms, Inc.), with the pendant drop method.^38,39^ The properties of the continuous phase, i.e., water plus Tween 80 in 1% concentration, considered herein at 25 °C were density ρ_*c*_ = 997.94 kg/m^3^ and the dynamic viscosity μ_*c*_ = 0.000927 kg/(m·s).

**Table 1:** Properties at 25 °C for the Oils Used

property	paraffin	soybean oil	isopropyl myristate	sunflower oil
density (ρ_*d*_) [kg/m^3^]	840.75	915.99	849.52	914.36
dynamic viscosity (μ_*d*_) [kg/(m·s)]	0.0320	0.0597	0.0050	0.0628
interfacial tension in water with 1% Tween 80 (γ) [mN/m]	9.97	6.91	6.99	6.42

#### Emulsion Characterization

The emulsions obtained were characterized by DSD and *d*_32_, which were determined by the laser diffraction method using Malvern Mastersizer-3000 (Malvern Instruments Ltd., Malvern, UK); the procedure was similar (with slight modifications) to that adopted by Ushikubo and Cunha.^35^ The emulsions were dispersed in deionized water. The measurements were conducted at laser obscuration between 10 and 12%, and the rotational speed of the circulating pump was set to 1800 rpm. The refractive index and the absorption of the aqueous phase were 1.344 (measured by an Abbe refractometer) and 0.01, respectively, and the droplet size values were linked to the corresponding volume fractions. Each experiment was performed in triplicate, and deviations between the results obtained under the same conditions were less than 2%; the averages were eventually evaluated and reported. The droplets were observed by using an optical microscope (model BX50; Olympus) to investigate the morphology. These characterizations were performed immediately after the production and after 14 days to investigate the stability at room temperature.

### Mathematical Modeling

#### Kolmogorov’s Theory-Based Models

One of the earliest and most well-known correlations for predicting *d*_32_ was made by Shinnar and Church,^40^ employing the drop breakage criterion proposed by Hinze.^16^ The model is based on Kolmogorov’s theory of turbulence, which is valid within the turbulent inertial subrange. Provided that viscous resistance to drop breakup is negligible, the interfacial tension acting on the surface of oil droplets is balanced by the turbulence kinetic energy of the surrounding continuous phase;^16^ see Figure S2 which shows the kinetic energy power spectrum together with a sketch of the underlying Richardson’s cascade process.^41^ This equilibrium is mathematically expressed in eq 3. The spatially averaged left-hand-side represents the kinetic energy transferred from the surrounding flow velocity fluctuation, where ρ_*c*_ is the density of water and δ_*d*_*u* is the local fluid velocity variation on the droplet surface. The right-hand-side represents the interfacial tension on the droplet surface, where γ is the interfacial tension coefficient of the oil in water and *d* is the droplet diameter
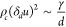
3

Kolmogorov’s 4/5-theory^41^ is one of the very few exact results in turbulence and states that in ideal turbulence, there exists a range of scales (the so-called inertial range) characterized by a constant flux of kinetic energy. Rephrasing it in a mean field sense (i.e., forgetting the minus sign telling us that energy transfers from large to small scales and omitting ensemble averages), the 4/5-law reads
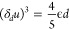
4where ϵ is the turbulent kinetic energy dissipation rate, has the same order of
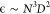
5where *N* is the rotational speed and *D* is the characteristic length, which in this case corresponds to the rotor diameter.^40,47^ By substituting eqs 4 and 5 into 3, the Sauter average diameter *d*_32_ is given as
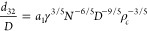
6where *a*_1_ is a constant coefficient determined from experimental data. Equation 6 can be recast in the following dimensionless form (attributed from here on to Shinnar and Church^40^)
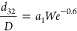
7where the Weber number is defined as
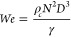
8

Note that the model given by eq 7 is valid only for dilute and noncoalescing systems (i.e., emulsions characterized by a very low volume fraction of the dispersed phase, ϕ_*v*_ → 0) and under the assumption of a negligible role of the oil viscosity, μ_*d*_, in the drop breakup mechanism (i.e., interfacial tension is the dominant resisting force).

In systems with a moderately higher content of the dispersed phase, for instance, 0.01 ≤ ϕ_*v*_ ≤ 0.3, that can still be described as noncoalescing, the role of oil concentration should, however, be taken into account since the neighboring drops start to alter the turbulence structure, affect small-scale turbulent fluctuations, and thus interfere with the drop breakage physics.^27^ A modified version of eq 7 accounting for the effect of the oil volume concentration is given by^18,19,25^
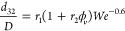
9where *r*_1_ and *r*_2_ are empirical constants.

Returning to dilute systems (ϕ_*v*_ → 0), one may adopt a complete mechanistic model able to capture the role of both the dispersed phase viscosity and the interfacial tension in resisting drop breakage due to the disruptive force (related to turbulent/inertial stress).^24^ Employing a basic mechanistic model, Calabrese and collaborators^21,42^ derived the following expression for the normalized Sauter mean diameter (here, we attribute this equation to Wang and Calabrese,^42^ who wrote it in this form by combining separate relations provided earlier by Calabrese et al.^21^)
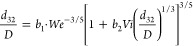
10where *b*_1_ and *b*_2_ are constants and *Vi* is the viscosity group defined as
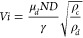
11

In the limiting case of *Vi* → ∞, i.e., negligible interfacial-tension resistance compared to the viscous resistance, eq 10 reduces to
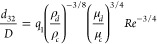
12which was first introduced by Calabrese et al.^21^ In eq 12, *q*_1_ is a constant and *Re* is the Reynolds number defined as
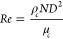
13

A more sophisticated mechanistic model taking into account the duration of the disruptive stress causing the deformation is based on representing the process with a Kelvin/Voigt element (a Hookean spring representing the interfacial tension effect connected in parallel to a Newtonian dashpot that models the viscous force).^24^ Following this approach, Das^43^ derived an expression for the maximum stable diameter of the drops (*d*_max_) in the turbulent inertial regime; however, a corresponding expression may be written for the Sauter mean diameter (*d*_32_), assuming proportionality between the two sizes.^21,27^ Accordingly, the model by Das^43^ yields
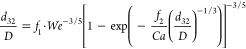
14where *f*_1_ and *f*_2_ are constants and *Ca* is the capillary number defined as
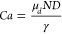
15

#### Present Π-Theorem-Based Model

The analytical model proposed here is based on the Buckingham Π-theorem,^44^ which states that the number of dimensionless groups, *p*, that define a problem equals the total number of variables, *n*, minus the number of fundamental dimensions, *k*. The dimensional form of the Sauter diameter can be written as
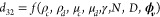
16

In eq 16, we skip the dependence on ρ_*d*_ since the density difference between oil and water is marginal and the role of buoyancy in the droplet breakup can be neglected. In addition, we assume that a concentration-weighted viscosity is sufficient to describe the role of the viscous effects in the mixing process; it is defined as
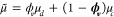
17

Equation 16 can therefore be simplified to
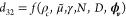
18

Since there are seven variables involved in this case and three fundamental dimensions, namely, mass, length, and time, we need to derive four dimensionless terms (Π groups). The latter can be written as the new functional relation
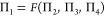
19

Applying the dimensional analysis, we obtain an explicit expression for eq 19 as
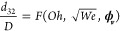
20where *Oh* is the Ohnesorge number, defined as
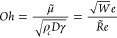
21with , the modified Reynolds number. In eq 20, the relation between the inertial force acting on the droplets and the interfacial tension is given by the Weber number, while the relation of the viscous force, inertial force, and interfacial tension is given by the Ohnesorge number. The validity of excluding the oil density and using the concentration-weighted viscosity will be attested to by comparing the model results with experimental data. The correlation is assumed to have the form
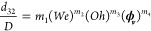
22where the constant *m*_1_ and the exponents *m*_2_, *m*_3_, and *m*_4_ are to be determined through the fitting of experimental data. In particular, the generalized reduced gradient (GRG) method^45^ was used to determine the coefficients of eq 22 and was efficiently combined with the Global Newton method for solving the equation system. The relative deviation of the model prediction relative to the experimental value for each sample was expressed as follows
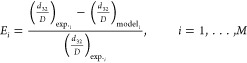
23where *M* corresponds to the number of samples.

The overall deviation of the model predictions was estimated in terms of both the maximum absolute error (*E*_max_) and the root-mean-square error (*E*_rms_); they are defined as
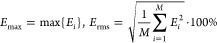
24

The objective of regression was initially to minimize *E*_rms_, yet it was found that minimizing *E*_max_ instead is able to enhance the predictions without considerably compromising the minimum *E*_rms_ obtained.

## Results and Discussion

### Emulsion Production

Granulometric data of the obtained emulsions are reported in terms of the Sauter mean diameter *d*_32_ and the representative diameters *d*_10_^a^, *d*_50_, *d*_90_, see Table S2. Paraffin oil and soybean oil as dispersed phases produce similar range of Sauter diameters, 16.20–4.35 and 12.00 and 2.74 μm, respectively. Smaller drops were obtained for isopropyl myristate-based emulsion; 6.71–1.83 μm.

**Figure 1:**
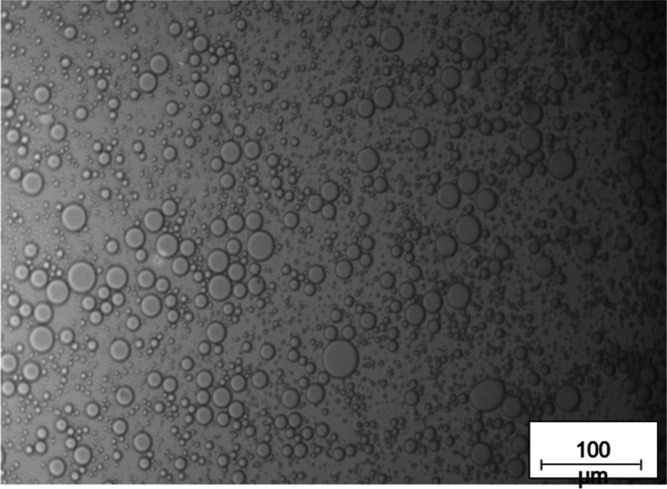
Representative optical microscope images of emulsion obtained with soybean oil as disperse phase with ϕ_*m*_ = 0.2 and a rotor speed of 5000 rpm. Scale bar = 100 μm. Images for all tests at 5000 rpm are given in Figure S3.

**Figure 2:**
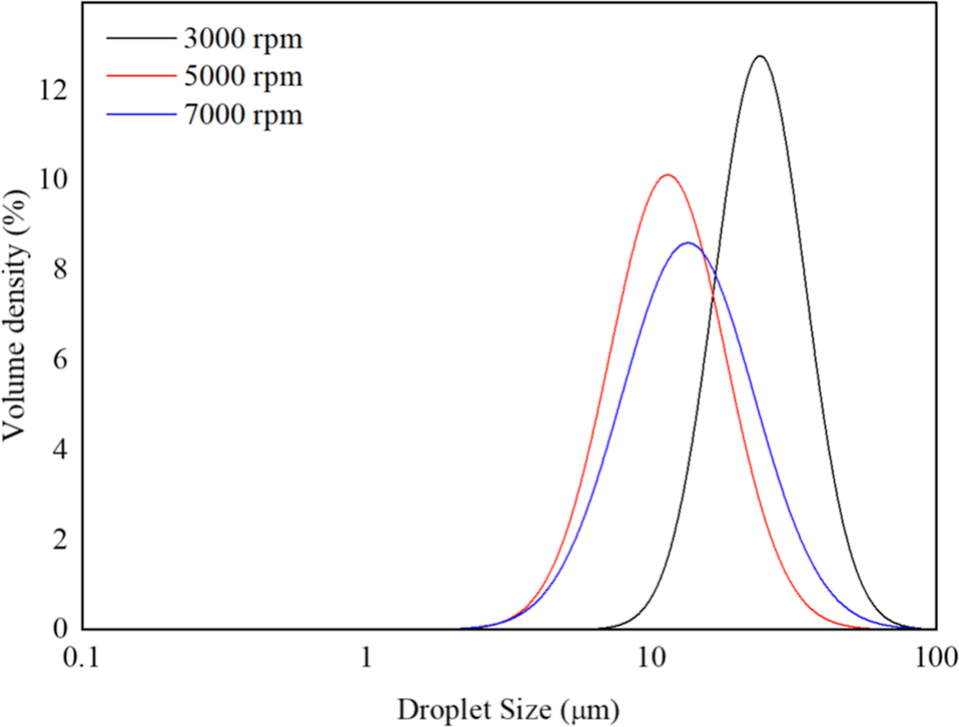
Droplet size distributions of emulsion obtained with soybean oil at different rotor speeds with ϕ_*m*_ = 0.2. Images for all tests with ϕ_*m*_ = 0.2 are given in Figure S4.

**Figure 3:**
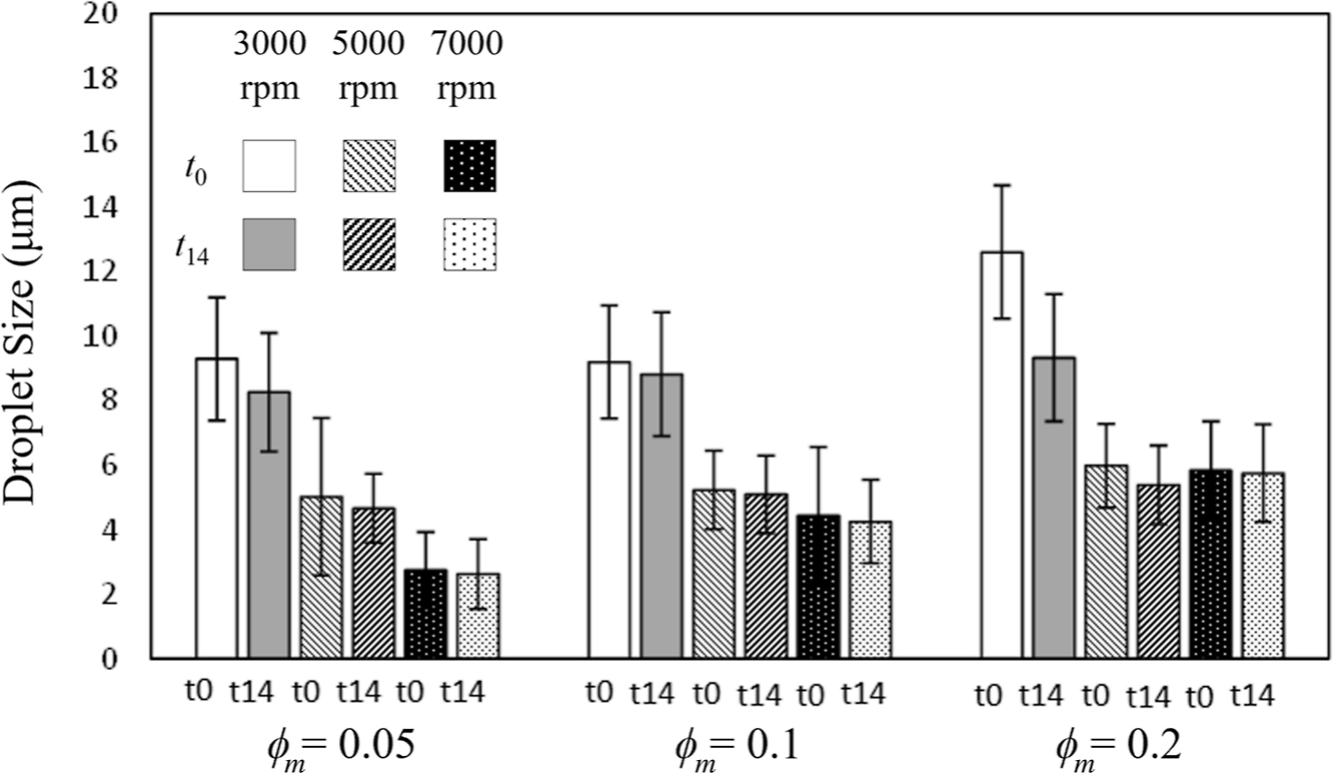
Distributions of *d*_32_ for emulsions obtained with soybean oil at different rotor speeds (3000, 5000, and 7000 rpm) and different oil concentrations (ϕ_*m*_ = 0.05, 0.1, and 0.2) at the initial time (*t*_0_) and after 14 days (*t*_14_). Images for all tests are given in Figure S5.

Produced emulsions were characterized by their opaque appearance and well-formed noncoalescing droplets, which can be observed in Figure 1, where optical microscope images of emulsions obtained for soybean oil with ϕ_*m*_ = 0.2 and a rotor speed of 5000 rpm. From Figure S3, one can observe that reducing the O/W ratio leads to the production of a more dilute emulsion. Droplet size distributions for soybean oil at different rotor speeds are listed in Figure 2. As shown in Figure S4, the mean diameter for the oils generally decreases with increasing rotor speed. All the tested oils present a monomodal dispersion at all the rotor speeds investigated. After 14 days from the emulsion production, the mean diameter of the drops was reanalyzed to verify their stability at room temperature. As reported in Figure 3, the emulsion from soybean oil showed good stability after 14 days at room temperature; the mean diameter did not increase. This implies that instability mechanisms such as separation, flocculation, coalescence, and phase inversion have been avoided.^46^ The same is true for the other oils investigated; see Figure S5.

### Sauter Mean Diameter Estimations with Theoretical Models

The theoretical models presented by Shinnar and Church^40^ (eq 7), Wang and Calabrese^42^ (eq 10), and Das^43^ (eq 14), in addition to the general form of models considering oil concentration (eq 9) and a simplified model (eq 12), as proposed by Calabrese et al.,^21^ were evaluated and compared to the newly proposed model (eq 22). The GRG method was applied for all of the correlations considered, both our new and literature models, to obtain unique coefficients/exponents for each model to best fit the experimental values of the normalized Sauter mean diameter. The dimensionless parameters are given in Table S3, the different models with computed coefficients/exponents are shown in Table S4, and the corresponding diameters are shown in Table S5.

**Figure 4:**
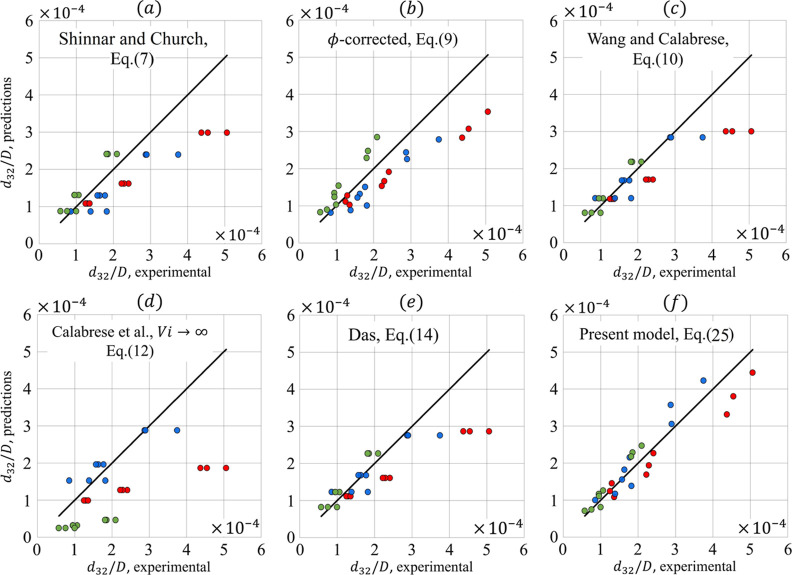
Scatter plots for *d*_32_/*D* predictions (all oils considered) made based on the experimental data against (a) Shinnar and Church, (b) ϕ – *corrected*, (c) Wang and Calabrese, (d) Calabrese et al., (e) Das, and (f) the proposed model. Paraffin, soybean oil, and isopropyl myristate experiments are represented by the red, blue, and green colored circles, respectively.

**Figure 5:**
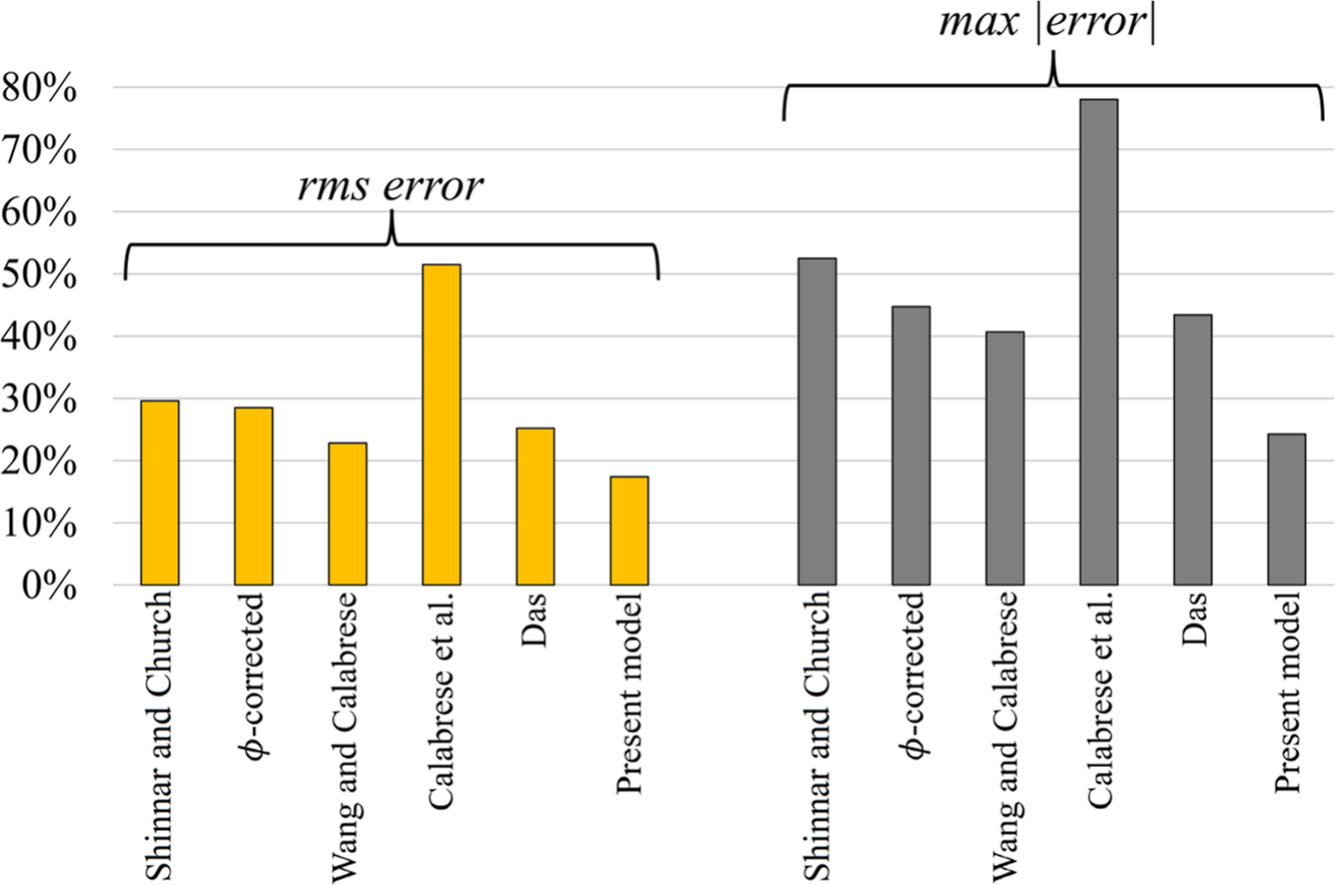
The rms errors and the maximum absolute deviations obtained by the assessed models, based on the optimized fittings for all oils together, in correspondence to Figure 4.

Figure 4 shows scatter plots for all models, derived using the results of all oils against the experimental results. For the present model, the fit with the theoretical line is reasonable and consistent for all diameters. The corresponding root-mean-square (RMS) and max errors are given in Figure 5. For both measures, the proposed model presents the lowest errors. In comparison with the literature models, see Table S4, we can first of all note that the proposed model is the only one accounting for *We*, *Oh*, and ϕ_*v*_, simultaneously. All models except the ϕ-corrected one exclude the oil volume concentration. This results in less accurate predictions for the larger drop diameters. The largest RMS and maximum absolute errors are found for the simplified model (Calabrese et al.). This is probably attributed to the absence of an interfacial tension in the model. Despite being elegant, the model by Shinnar and Church^40^ lacks both the oil concentration and the viscosity, which results in rather large RMS errors. Accordingly, we can conclude that the introduced Π-theorem-based correlation, including simultaneously *We*, *Oh*, and ϕ_*v*_, has shown its validity in comparison with theories based on the 4/5-law of Kolmogorov and Kelvin/Voigt-based models. It explicitly describes the Sauter mean diameter, and it may be adjusted to fit conditions other than the turbulent inertial subregime being considered. The explicit expression for the introduced correlation, accounting for all oils, is given as
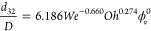
25valid within the following ranges (see also Table S3)
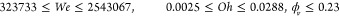
26

Despite the fact that the fitting exponent of ϕ_*v*_ in eq 25 is *zero*, it is important to emphasize that the oil volume concentration, ϕ_*v*_, is still contributing to our model, implicitly in the Ohnesorge number, *Oh*, through the definition of the weighted viscosity μ. Scrutinizing the new model, we note that the *Oh*-term has significant contribution to the normalized Sauter diameter. One explanation might be that hydrodynamics is important, and this is related to the Reynolds number (*Re*), which varies from 3 × 10^5^ to 8 × 10^5^ here. Theoretically, we see this by writing  (roughly, since *Oh* is formally defined in this work based on the modified Reynolds number), such that it becomes evident that *Re* is the main difference between *Oh* and *We*. Finally, the correlation coefficients should be recalibrated if the operative conditions are out of the previous ranges and/or the mixer geometry is different.

### Model Validation

The new model (eq 25) was further validated with two additional sets of experimental data, which were not used during the calibration of its coefficients and exponents. The first experimental set was made with isopropyl myristate oil subjected to three different surfactants. In particular, Tween 80, Pluronic, and PVA were used with concentrations of 0.5, 1, and 1.5%, respectively. The interfacial tension of isopropyl myristate in water with different surfactant concentration and the corresponding water viscosity, density, and surface tension in air are shown in Table S6. The reported values are measured with the same instruments and procedures already described in the Materials Section. Moreover, the model was also validated against experimental results using sunflower oil exposed to different rotating speeds. All validation results are shown in Table S7. The RMS errors are generally larger compared to those obtained during the calibration. This might be due to the surfactants not being explicitly part of the model but implicitly accounted for in density and interfacial tension values. However, the proposed model has the lowest error with respect to experimental values when compared to the literature models tested herein.

## Conclusions

Following an experimental/analytical framework, this paper explores the rich literature on emulsion characterization and analysis and eventually yields a novel correlation for the prediction of the Sauter mean droplet size. First, 27 experiments on O-W emulsions produced by a rotor-stator mixer have been conducted using three oils (paraffin, soybean oil, and isopropyl myristate), at different rotor speeds (3000–5000–7000 rpm), and with different oil mass concentrations in the emulsion (ϕ_*m*_ = 0.2–0.1–0.05). The droplet size distributions and mean droplet diameters were measured at the initial time and after 14 days to verify the stability of emulsions at room temperature. Then, a general Π-theorem-based correlation of the Sauter mean diameter was formulated, and the experimentally measured Sauter mean droplet diameters of stable emulsions were employed to obtain the correlation constants via multiple regression analysis. Accordingly, the Sauter mean diameter (normalized by the rotor workhead diameter) was explicitly expressed as a function of the Weber number (*We*), the Ohnesorge number (*Oh*), and the oil volume concentration (ϕ_*v*_). In other words, the proposed expression links the Sauter mean diameter (*d*_32_) to the rotor diameter (*D*) and speed (*N*), the water density (ρ_*c*_), the oil viscosity (μ_*d*_), the water viscosity (μ_*c*_), and the interfacial tension coefficient (γ), besides the role of oil volume concentration. The proposed model showed the best agreement compared to the literature models tested. The RMS deviation between the model predictions for *d*_32_ and the full set of experimental results was estimated to be 17.4%, compared to RMS deviations of 29.58% for the model by Shinnar and Church,^40^ 22.80% for the model by Wang and Calabrese,^42^ and 25.18% for the model by Das.^43^ The eminence of the proposed model is probably due to the fact that it simultaneously accounts for *We*, *Oh*, and ϕ_*v*_, while the other models account for two or less of the before-mentioned nondimensional numbers. An additional set of experimental results, using isopropyl myristate with three different surfactants as well as sunflower oil, was produced just for validation purposes. The RMS error for the proposed model was larger but still lower than those obtained with the literature models just cited. The larger error might be due to surfactants not being contained explicitly in the model but implicitly in the density and interfacial tension values. Note that the values for all densities and interfacial tensions used in this work have been carefully measured by ourselves. The results show that the proposed model is robust in the parameter range tested and is an improvement compared to a number of models already present in the literature. Future developments of the present work will be the testing of the model on different emulsion-based systems in order to extend the applicability of the proposed model and refine its accuracy in a wide range of systems of interest for different fields of application. Furthermore, since in many processes, emulsions are used as templates for particle production, a possible future application of the proposed model will be the prediction of particle mean diameters obtained starting from emulsions.

## Supporting Information Available

Rotor-stator emulsifier, turbulent energy spectrum, optical microscope images of emulsions at 5000 rpm, droplet size distributions for ϕ_*m*_ = 0.2, *d*_32_ distributions at initial time and after 14 days, models of Sauter diameter, experimental values of representative diameters, dimensionless numbers for all experimental conditions, tested models with optimized coefficients, Sauter averaged diameter for all oils, measured water properties and interfacial tension of isopropyl myristate with different surfactants, and *d*_32_/*D* for all validation cases (PDF)

## Footnotes

a 10% of the particles have diameters smaller than the threshold *d*_10_.
