# Supplementary material for: Rotor-Stator Emulsification in the Turbulent Inertial Regime: Experiments toward a Robust Correlation for the Droplet Size
Source: Langmuir. 2023 Dec 6;39(50):18518–25. doi: 10.1021/acs.langmuir.3c02868 (PMC10734302; doi:10.1021/acs.langmuir.3c02868)
Supplement: Supplementary file 1 — la3c02868_si_001.pdf [file la3c02868_si_001.pdf]

# Supporting Information: On rotor-stator emulsification in the turbulent inertial regime: Experiments toward a robust correlation for the droplet size

Roberta Campardelli[1,†] Giulia De Negri Atanasio[1,†] Claudia Carotenuto[2,‡] Raffaella Griffo[2,‡] Essam Nabil Ahmed[1,†] Manuel Corrales-González[1,†] Jiasen Wei[1,†] Peter Enos Tuju[1,†] Andrea Mazzino[1,†] and Jan Oscar Pralits[1*,†]

†[1]*Dep. of Civil, Chemical and Environmental Engineering, University of Genoa, Genoa, Italy*

‡[2]*Dep. of Engineering, University of Campania "Luigi Vanvitelli", Aversa (Caserta) Italy.*

E-mail: jan.pralits@unige.it

**List of content**

Rotor-stator emulsifier (Figure S1), turbulent energy spectrum (Figure S2), optical microscope images of emulsions at 5000 rpm (Figure S3), droplet size distributions for $\phi_m = 0.2$ (Figure S4), $d_{32}$ distributions at initial time and after 14 days (Figure S5), models of Sauter diameter (Table S1), experimental values of representative diameters (Table S2), dimensionless numbers for all experimental conditions (Table S3), tested models with optimized coefficients (Table S4), Sauter averaged diameter for all oils (Table S5), measured water properties and interfacial tension of isopropyl myristate with different surfactants (Table S6), $d_{32}/D$ for all validation cases (Table S7).

Table S1: Models available in the literature for droplet diameter of O-W emulsions in turbulence inertial sub-regime.

| Authors | Correlation | Theory/Equipment |
|---|---|---|
| Shinnar & Church (1960)[S1] | $d_{32}/D = a_1 \cdot We^{-0.6}$ | - Kolmogorov's theory, - Hinze's criterion[S2] for a negligible viscous stress |
| Brown & Pitt (1971)[S3] | $d_{32}/D = 0.051(1+3.14\phi_v) \cdot We^{-0.6}$ | Concentration-correction |
| Mlynek & Resnick (1972)[S4] | $d_{32}/D = 0.058(1+5.4\phi_v) \cdot We^{-0.6}$ | Concentration-correction |
| Calabrese et al. (1986a)[S5] | $d_{32}/D = q_1(\frac{\rho_d}{\rho_c})^{-3/8}(\frac{\mu_d}{\mu_c})^{3/4}Re^{-3/4}$ | Kolmogorov's theory, Interfacial tension neglected |
| Wang & Calabrese (1986)[S6] | $d_{32}/D = b_1 \cdot We^{-0.6}[1+b_2Vi(d_{32}/D)^{1/3}]^{3/5}$ | Full Mechanistic model (viscous and interfacial forces considered) |
| Nishikawa et al. (1987)[S7] | $d_{32}/D = 0.095N_p^{-0.4}We^{-0.6}(1+2.5\phi_v^{2/3})$ $(\mu_d/\mu_c)_d^{1/5}(\mu_d/\mu_c)_c^{1/8}$ | The suffix c and d means keeping the viscosity of water or oil as constant, respectively |
| Baldyga & Bourne (1992)[S8] | $d_{32}/D = z_1 \cdot We^{-0.6z_2}$ | The turbulence theory, the $\beta$ model |
| Das (1996)[S9] | $d_{32}/D = f_1 \cdot We^{-3/5}$ $[1-exp(-\frac{f_2}{Ca}(d_{32}/D)^{-1/3})]^{-3/5}$ | Kelvin/Voigt element-based mechanistic model |
| Hall et al. (2013)[S10] | $d_{32}/D = k_1\epsilon^{k_2}t_R^{k_3}$ | Rotor–stator mixer |
| Liu et al. (2013)[S11] | $d_{32}/D = p_1(1+\alpha\phi_v)[1+p_2(1+\alpha\phi_v)^{-5/6}/\gamma$ $(\rho_c/\rho_d^{1/2})\mu_dNL^{2/3}(d_{32}/D)^{1/3}]^{3/5}$ $N^{-6/5}L^{-4/5}\gamma^{3/5}\rho_c^{-3/5}$ | Rotor-stator mixer |

* Some models were originally written for the maximum droplet diameter ($d_{max}$). However, they are recast here in terms of the mean droplet size ($d_{32}$) based on the proportionality ($d_{max} \sim d_{32}$) in a diluted system.
* $a_1, b_1, b_2, z_1, z_2, q_1, f_1, k_1, k_2, k_3, p_1, p_2$, and $\alpha$ are constants.
* $\phi_v$ is the dispersion volume concentration. $\rho$ is the density. $\mu$ is the dynamic viscosity. The suffix $c$ and $d$ denote properties for water and oil, respectively. $\epsilon$ is the turbulence energy dissipation rate. $\gamma$ is the interfacial tension. $t_R$ is the total residence time for coalescence.
* $N$(rpm) is the rotation speed of the rotor-stator mixer. $D$ is the characteristic diameter, which is the diameter of the rotor head for a rotor-stator mixer. $L$ is the outer rotor diameter. $N_p$ is the power number.[S7]
* The dimensionless quantities: $Re = \rho_c ND^2/\mu_c$, $We = \rho_c N^2 D^3/\gamma$, $Vi = \sqrt{\frac{\rho_c}{\rho_d}}\mu_d ND/\gamma$, and $Ca = \mu_d ND/\gamma$.

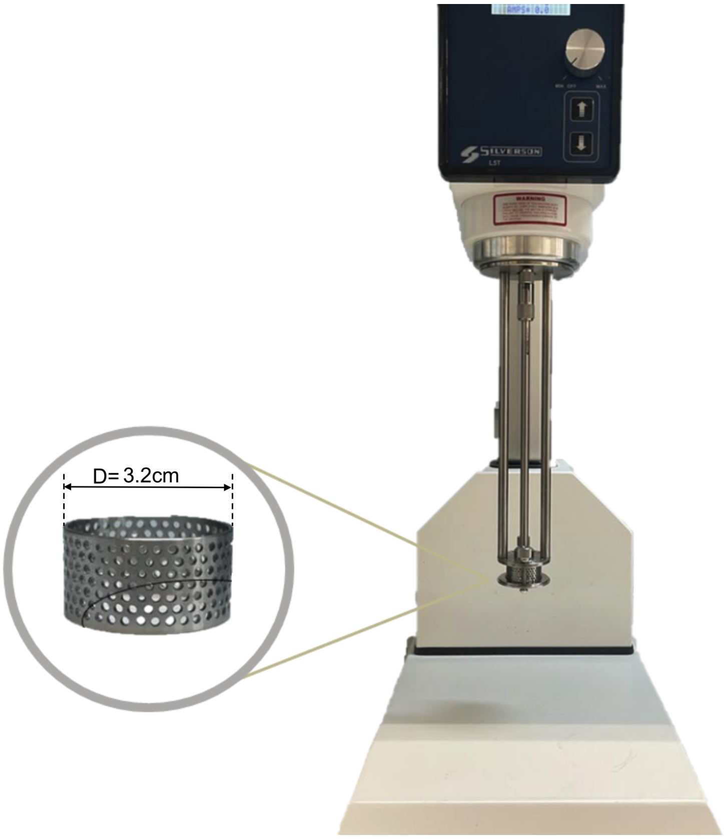


Figure S1: Representative image of the rotor-stator emulsifier and the used workhead.

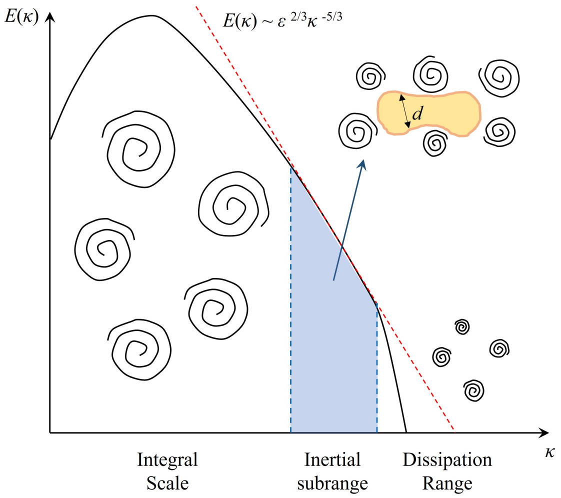


Figure S2: Sketch of Richardson regime and energy spectrum of turbulent flow in log-log scale.

Table S2: Experimental values of mean and representative diameters of emulsion drops.

| Oil | Rotational speed [rpm] | $\phi_m$ | $d_{32}$ [μm] | $d_{10}$ [μm] | $d_{50}$ [μm] | $d_{90}$ [μm] |
|---|---|---|---|---|---|---|
| Paraffin | 3000 | 0.2 | 16.20 | 17.30 | 29.60 | 44.35 |
| | | 0.1 | 14.55 | 16.60 | 28.40 | 42.30 |
| | | 0.05 | 14.00 | 15.40 | 26.40 | 39.20 |
| | 5000 | 0.2 | 7.71 | 7.25 | 13.55 | 20.50 |
| | | 0.1 | 7.32 | 6.53 | 12.80 | 20.30 |
| | | 0.05 | 7.11 | 6.10 | 12.60 | 19.50 |
| | 7000 | 0.2 | 4.16 | 2.15 | 6.29 | 12.30 |
| | | 0.1 | 4.00 | 1.95 | 6.40 | 12.55 |
| | | 0.05 | 4.35 | 2.04 | 7.26 | 13.30 |
| Soybean oil | 3000 | 0.2 | 12.00 | 12.6 | 24.80 | 38.55 |
| | | 0.1 | 9.19 | 7.63 | 19.70 | 32.80 |
| | | 0.05 | 9.29 | 4.71 | 21.80 | 36.95 |
| | 5000 | 0.2 | 5.68 | 2.48 | 10.90 | 19.25 |
| | | 0.1 | 5.23 | 2.16 | 10.40 | 18.90 |
| | | 0.05 | 5.02 | 2.12 | 9.26 | 19.85 |
| | 7000 | 0.2 | 5.83 | 2.43 | 12.75 | 25.20 |
| | | 0.1 | 4.43 | 1.68 | 10.40 | 24.15 |
| | | 0.05 | 2.74 | 0.96 | 5.93 | 16.60 |
| Isopropyl myristate | 3000 | 0.2 | 6.71 | 4.00 | 11.20 | 19.00 |
| | | 0.1 | 5.93 | 3.13 | 10.00 | 17.40 |
| | | 0.05 | 5.81 | 3.08 | 9.69 | 16.90 |
| | 5000 | 0.2 | 3.14 | 1.74 | 4.84 | 9.81 |
| | | 0.1 | 3.04 | 1.51 | 4.20 | 8.62 |
| | | 0.05 | 3.05 | 1.43 | 4.25 | 11.30 |
| | 7000 | 0.2 | 3.20 | 1.84 | 4.04 | 8.54 |
| | | 0.1 | 2.42 | 1.28 | 3.10 | 6.62 |
| | | 0.05 | 1.83 | 0.88 | 2.42 | 5.61 |

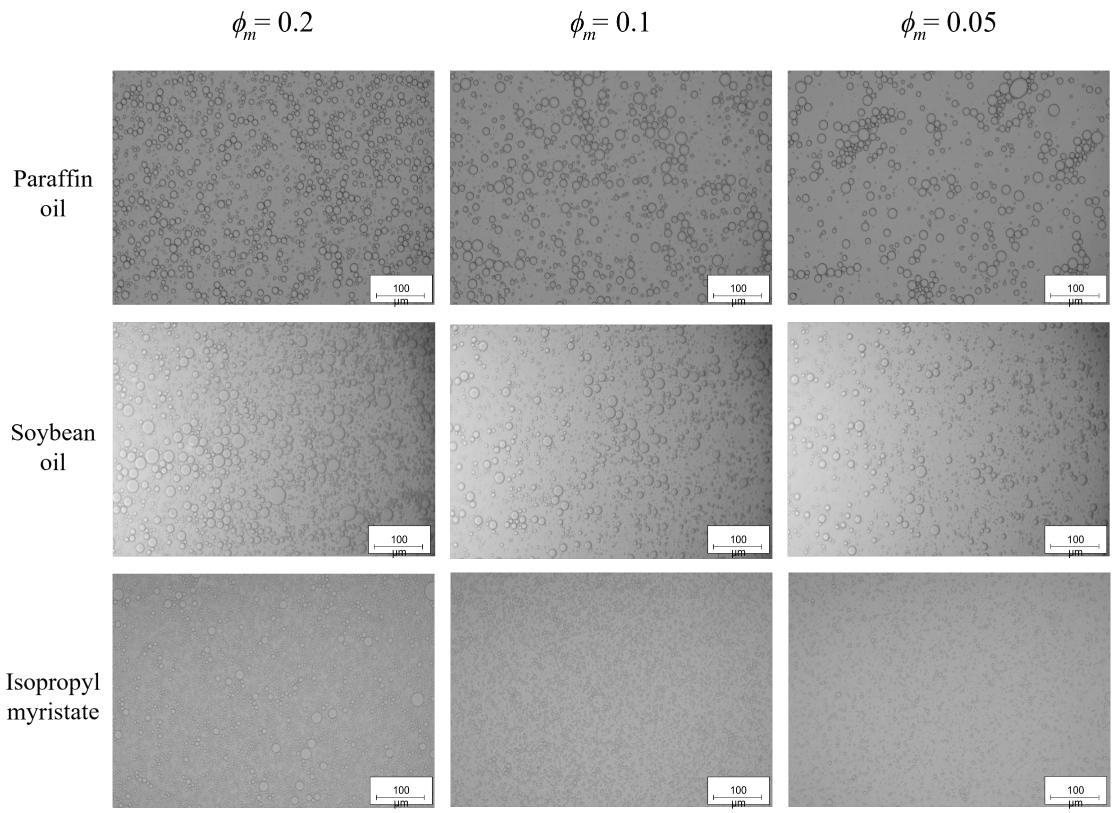


Figure S3: Representative optical microscope images of emulsions obtained with paraffin, soybean oil, and isopropyl myristate as disperse phases, as a function of the oil concentration at a fixed rotor speed of 5000 rpm. Scale bar 100 μm.

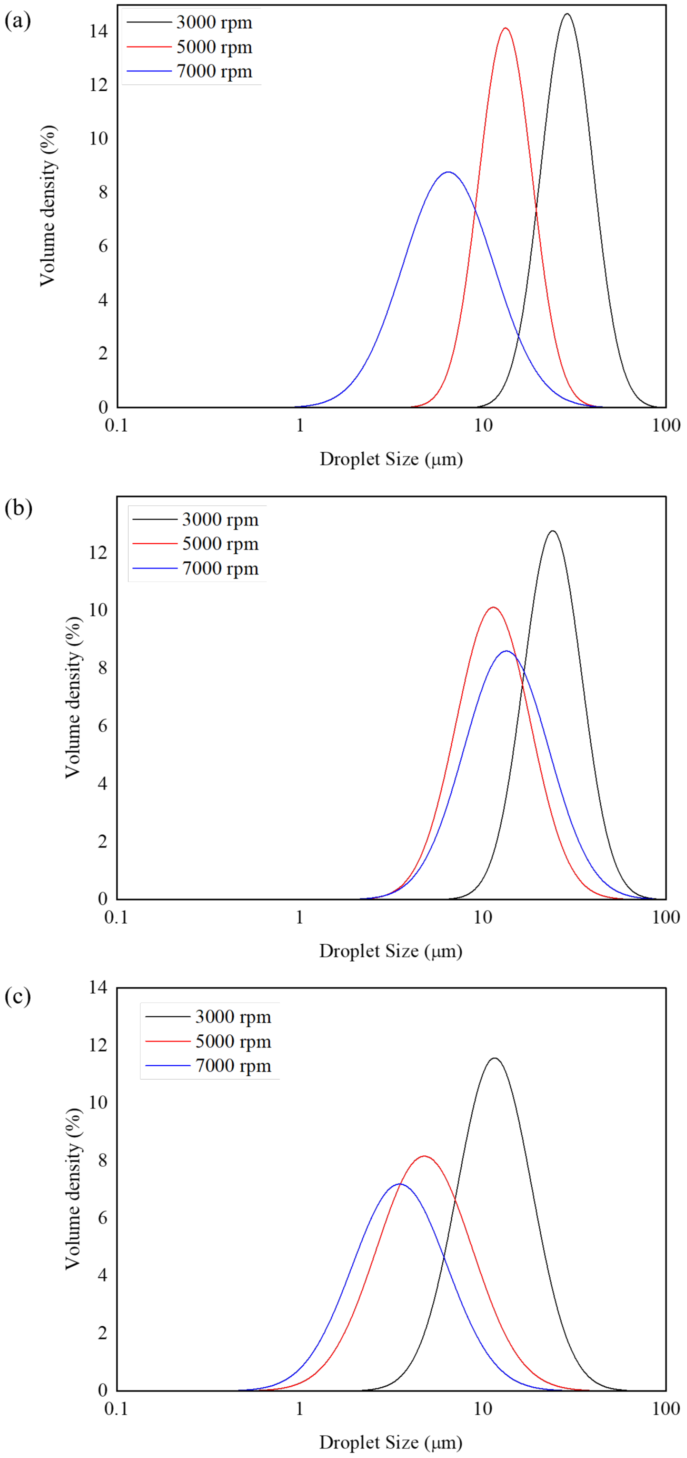


Figure S4: Droplet size distributions of emulsions obtained with (a) paraffin oil, (b) soybean oil, and (c) isopropyl myristate; at different rotor speeds with fixed representative oil concentration of $\phi_m = 0.2$.

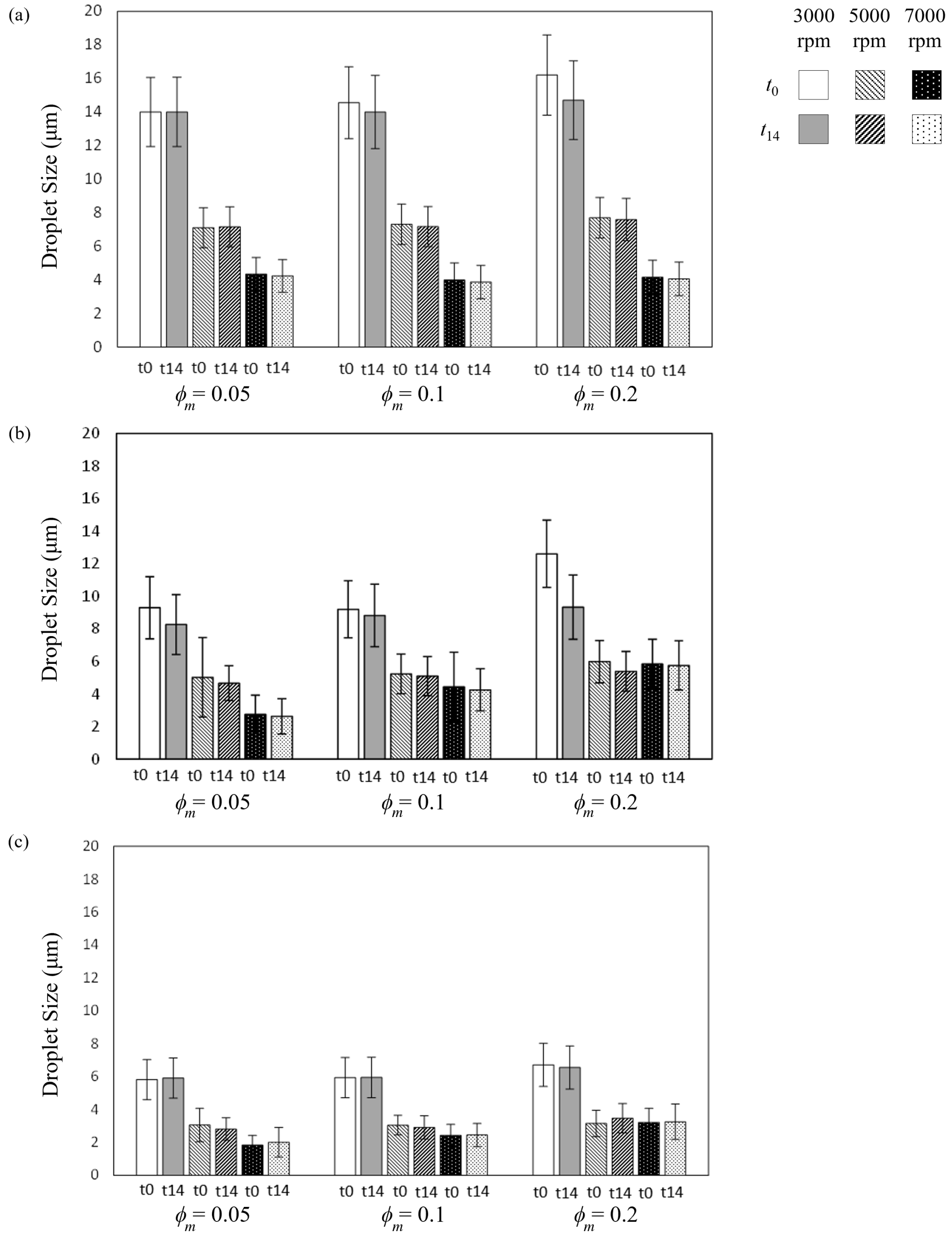


Figure S5: $d_{32}$ distributions of emulsions obtained with (a) paraffin, (b) soybean oil, and (c) isopropyl myristate, at different rotor speeds (3000, 5000 and 7000 rpm) and different oil concentrations ($\phi_m = 0.05, 0.1, 0.2$) at the initial time ($t_0$) and after 14 days ($t_{14}$).

Table S3: Dimensionless numbers for all the experimental conditions.

| Oil | $N$ [rpm] | $\phi_m$ | $\phi_v$ | Re | Ca | Vi | We | Oh | $d_{32}/D$, Exp |
|---|---|---|---|---|---|---|---|---|---|
| Paraffin | 3000 | 0.2 | 0.229 | 346339 | 32.267 | 35.155 | 323733 | 0.0142 | 0.000506 |
| | 3000 | 0.1 | 0.117 | 346339 | 32.267 | 35.155 | 323733 | 0.0081 | 0.000455 |
| | 3000 | 0.05 | 0.059 | 346339 | 32.267 | 35.155 | 323733 | 0.0049 | 0.000438 |
| | 5000 | 0.2 | 0.229 | 577232 | 53.778 | 58.592 | 899259 | 0.0142 | 0.000241 |
| | 5000 | 0.1 | 0.117 | 577232 | 53.778 | 58.592 | 899259 | 0.0081 | 0.000229 |
| | 5000 | 0.05 | 0.059 | 577232 | 53.778 | 58.592 | 899259 | 0.0049 | 0.000222 |
| | 7000 | 0.2 | 0.229 | 808125 | 75.289 | 82.028 | 1762547 | 0.0142 | 0.000130 |
| | 7000 | 0.1 | 0.117 | 808125 | 75.289 | 82.028 | 1762547 | 0.0081 | 0.000125 |
| | 7000 | 0.05 | 0.059 | 808125 | 75.289 | 82.028 | 1762547 | 0.0049 | 0.000136 |
| Soybean | 3000 | 0.2 | 0.214 | 346339 | 86.855 | 90.660 | 467094 | 0.0288 | 0.000375 |
| | 3000 | 0.1 | 0.108 | 346339 | 86.855 | 90.660 | 467094 | 0.0155 | 0.000287 |
| | 3000 | 0.05 | 0.054 | 346339 | 86.855 | 90.660 | 467094 | 0.0088 | 0.000290 |
| | 5000 | 0.2 | 0.214 | 577232 | 144.759 | 151.100 | 1297483 | 0.0288 | 0.000178 |
| | 5000 | 0.1 | 0.108 | 577232 | 144.759 | 151.100 | 1297483 | 0.0155 | 0.000164 |
| | 5000 | 0.05 | 0.054 | 577232 | 144.759 | 151.100 | 1297483 | 0.0088 | 0.000157 |
| | 7000 | 0.2 | 0.214 | 808125 | 202.663 | 211.541 | 2543067 | 0.0288 | 0.000182 |
| | 7000 | 0.1 | 0.108 | 808125 | 202.663 | 211.541 | 2543067 | 0.0155 | 0.000139 |
| | 7000 | 0.05 | 0.054 | 808125 | 202.663 | 211.541 | 2543067 | 0.0088 | 0.000086 |
| Isopropyl | 3000 | 0.2 | 0.227 | 346339 | 7.191 | 7.794 | 461748 | 0.0039 | 0.000210 |
| | 3000 | 0.1 | 0.115 | 346339 | 7.191 | 7.794 | 461748 | 0.0030 | 0.000185 |
| | 3000 | 0.05 | 0.058 | 346339 | 7.191 | 7.794 | 461748 | 0.0025 | 0.000182 |
| | 5000 | 0.2 | 0.227 | 577232 | 11.985 | 12.990 | 1282633 | 0.0039 | 0.000107 |
| | 5000 | 0.1 | 0.115 | 577232 | 11.985 | 12.990 | 1282633 | 0.0030 | 0.000095 |
| | 5000 | 0.05 | 0.058 | 577232 | 11.985 | 12.990 | 1282633 | 0.0025 | 0.000095 |
| | 7000 | 0.2 | 0.227 | 808125 | 16.779 | 18.186 | 2513962 | 0.0039 | 0.000100 |
| | 7000 | 0.1 | 0.115 | 808125 | 16.779 | 18.186 | 2513962 | 0.0030 | 0.000076 |
| | 7000 | 0.05 | 0.058 | 808125 | 16.779 | 18.186 | 2513962 | 0.0025 | 0.000057 |

Table S4: List of tested models with optimized coefficients/exponents

| Model | Equation |
|---|---|
| Shinnar and Church (1960) | $d_{32}/D = 0.604\, We^{-0.6}$ |
| $\phi$-corrected | $d_{32}/D = 0.524(1 + 1.583\, \phi_v) We^{-0.6}$ |
| Wang and Calabrese (1986) | $d_{32}/D = 0.530\, We^{-0.6}[1 + 0.109 Vi\, (d_{32}/D)^{1/3}]^{3/5}$ |
| Calabrese et al. (1986a), $Vi \rightarrow \infty$ | $d_{32}/D = 0.175(\frac{\rho_d}{\rho_c})^{-3/8}(\frac{\mu_d}{\mu_c})^{3/4} Re^{-3/4}$ |
| Das (1996) | $d_{32}/D = 0.567\, We^{-3/5}[1 - exp(-\frac{7.051}{Ca}(d_{32}/D)^{-1/3})]^{-3/5}$ |
| Proposed model | $d_{32}/D = 6.186\, We^{-0.660}\, Oh^{0.274}\, \phi_v^0$ |

Table S5: Estimation of normalized Sauter averaged diameter for all oils.

| | Experimental Data | | | $d_{32}/D \times 10^{-4}$ | | | | | |
|---|---|---|---|---|---|---|---|---|---|
| Oil | $\phi_v$ | $N$ [rad/s] | $d_{32}/D \times 10^{-4}$ | Shinnar & Church (1960) | $\phi$-correlated | Wang & Calabrese (1986) | Calabrese et al. (1986a), for $Vi \to \infty$ | Das (1996) | Present model Π-theory |
| Paraffin | 0.229 | 314.16 | 5.063 | 2.983 | 3.531 | 3.004 | 1.862 | 2.865 | 4.443 |
| | 0.117 | 314.16 | 4.547 | 2.983 | 3.070 | 3.004 | 1.862 | 2.865 | 3.801 |
| | 0.059 | 314.16 | 4.375 | 2.983 | 2.833 | 3.004 | 1.862 | 2.865 | 3.314 |
| | 0.229 | 523.6 | 2.409 | 1.616 | 1.913 | 1.702 | 1.269 | 1.606 | 2.263 |
| | 0.117 | 523.6 | 2.288 | 1.616 | 1.663 | 1.702 | 1.269 | 1.606 | 1.936 |
| | 0.059 | 523.6 | 2.222 | 1.616 | 1.535 | 1.702 | 1.269 | 1.606 | 1.688 |
| | 0.229 | 733.04 | 1.300 | 1.079 | 1.277 | 1.178 | 0.986 | 1.112 | 1.451 |
| | 0.117 | 733.04 | 1.250 | 1.079 | 1.111 | 1.178 | 0.986 | 1.112 | 1.242 |
| | 0.059 | 733.04 | 1.359 | 1.079 | 1.025 | 1.178 | 0.986 | 1.112 | 1.082 |
| Soybean | 0.214 | 314.16 | 3.750 | 2.394 | 2.785 | 2.838 | 2.878 | 2.755 | 4.228 |
| | 0.108 | 314.16 | 2.872 | 2.394 | 2.436 | 2.838 | 2.878 | 2.755 | 3.569 |
| | 0.054 | 314.16 | 2.900 | 2.394 | 2.259 | 2.838 | 2.878 | 2.755 | 3.053 |
| | 0.214 | 523.6 | 1.777 | 1.297 | 1.509 | 1.678 | 1.962 | 1.678 | 2.154 |
| | 0.108 | 523.6 | 1.636 | 1.297 | 1.319 | 1.678 | 1.962 | 1.678 | 1.818 |
| | 0.054 | 523.6 | 1.570 | 1.297 | 1.224 | 1.678 | 1.962 | 1.678 | 1.555 |
| | 0.214 | 733.04 | 1.823 | 0.866 | 1.008 | 1.199 | 1.524 | 1.228 | 1.381 |
| | 0.108 | 733.04 | 1.386 | 0.866 | 0.881 | 1.199 | 1.524 | 1.228 | 1.166 |
| | 0.054 | 733.04 | 0.856 | 0.866 | 0.817 | 1.199 | 1.524 | 1.228 | 0.997 |
| Isopropyl | 0.227 | 314.16 | 2.097 | 2.411 | 2.847 | 2.181 | 0.461 | 2.264 | 2.468 |
| | 0.115 | 314.16 | 1.853 | 2.411 | 2.477 | 2.181 | 0.461 | 2.264 | 2.285 |
| | 0.058 | 314.16 | 1.816 | 2.411 | 2.288 | 2.181 | 0.461 | 2.264 | 2.174 |
| | 0.227 | 523.6 | 1.066 | 1.306 | 1.542 | 1.194 | 0.314 | 1.227 | 1.257 |
| | 0.115 | 523.6 | 0.950 | 1.306 | 1.342 | 1.194 | 0.314 | 1.227 | 1.164 |
| | 0.058 | 523.6 | 0.953 | 1.306 | 1.239 | 1.194 | 0.314 | 1.227 | 1.107 |
| | 0.227 | 733.04 | 1.000 | 0.872 | 1.030 | 0.804 | 0.244 | 0.819 | 0.806 |
| | 0.115 | 733.04 | 0.756 | 0.872 | 0.896 | 0.804 | 0.244 | 0.819 | 0.746 |
| | 0.058 | 733.04 | 0.572 | 0.872 | 0.828 | 0.804 | 0.244 | 0.819 | 0.710 |
| | | | $E_{rms}$ | 29.58% | 28.51% | 22.80% | 51.49% | 25.18% | 17.40% |
| | | | $E_{max}$ | 52.50% | 44.75% | 40.66% | 78.02% | 43.42% | 24.26% |

Table S6: Water properties with different surfactants, in addition to corresponding values of interfacial tension for isopropyl myristate in water.

| Surfactants | Concentration in water [%] | Density [kg/m$^3$] | Viscosity [mPa.s] | Surface tension in air [mN/m] | interfacial tension [mN/m] for isopropyl myristate |
|---|---|---|---|---|---|
| Tween 80 | 0.5 | 997.45 | 0.916 | 41.56 | 6.86 |
| | 1.0 | 997.94 | 0.927 | 41.59 | 6.99 |
| | 1.5 | 998.35 | 0.942 | 41.63 | 6.76 |
| Pluronic | 0.5 | 997.77 | 0.9883 | 43.35 | 12.95 |
| | 1.0 | 998.51 | 1.1001 | 41.61 | 12.53 |
| | 1.5 | 999.24 | 1.2248 | 40.87 | 11.71 |
| PVA | 0.5 | 998.18 | 1.0993 | 48.52 | 13.77 |
| | 1.0 | 999.25 | 1.3775 | 47.33 | 13.19 |
| | 1.5 | 1000.6 | 1.7204 | 47.23 | 12.19 |

Table S7: Estimation of $d_{32}/D$ for sunflower oil and isopropyl myristate ($\phi_m = 0.2$) with different surfactants in comparison with experiments.

| Oil | Surfactants | $N$ [rpm] | $d_{32}/D \times 10^{-4}$ | | | | | | |
|---|---|---|---|---|---|---|---|---|---|
| | | | Experiments | Shinnar & Church (1960) | $\phi$-corrected | Wang & Calabrese (1986) | Calabrese et al. (1986a), for $Vi \to \infty$ | Das (1996) | Present model $\Pi$-theory |
| Sunflower Oil | Tween 1% | 2000 | 7.219 | 3.729 | 4.333 | 4.276 | 4.054 | 4.112 | 7.062 |
| | Tween (1%) | 5000 | 2.366 | 1.242 | 1.443 | 1.665 | 2.039 | 1.685 | 2.107 |
| | Tween 1% | 9000 | 1.566 | 0.613 | 0.713 | 0.939 | 1.312 | 0.993 | 0.970 |
| Isopropyl myristate | Tween (0.5%) | 7000 | 1.175 | 0.863 | 1.018 | 0.796 | 0.244 | 0.810 | 0.800 |
| | Tween (1%) | | 1.034 | 0.873 | 1.029 | 0.804 | 0.244 | 0.819 | 0.809 |
| | Tween (1.5%) | | 0.888 | 0.855 | 1.009 | 0.789 | 0.244 | 0.803 | 0.796 |
| | Pluronic (0.5%) | | 0.603 | 1.263 | 1.490 | 1.143 | 0.244 | 1.186 | 1.124 |
| | Pluronic (1%) | | 0.488 | 1.238 | 1.460 | 1.121 | 0.244 | 1.162 | 1.118 |
| | Pluronic (1.5%) | | 1.066 | 1.188 | 1.402 | 1.078 | 0.244 | 1.116 | 1.093 |
| | PVA (0.5%) | | 0.713 | 1.311 | 1.546 | 1.184 | 0.244 | 1.230 | 1.175 |
| | PVA (1%) | | 1.209 | 1.276 | 1.506 | 1.160 | 0.244 | 1.205 | 1.180 |
| | PVA (1.5%) | | 1.059 | 1.216 | 1.435 | 1.109 | 0.244 | 1.149 | 1.167 |
| | | | $E_{rms}$ (%) | 66.07% | 83.88% | 54.08% | 63.48% | 57.29% | 51.39% |

# References


(S1) Shinnar, R.; Church, J. M. Statistical theories of turbulence in predicting particle size in agitated dispersions. *J Ind. Eng. Chem.* **1960**, *52*, 253–256.

(S2) Hinze, J. O. Fundamentals of the hydrodynamic mechanism of splitting in dispersion processes. *AIChE J.* **1955**, *1*, 289–295.

(S3) Brown, D. R.; Pitt, K. Drop break-up in a stirred liquid-liquid contactor. British Chem. Eng. 1971; p 525.

(S4) Mlynek, Y.; Resnick, W. Drop sizes in an agitated liquid-liquid system. *AIChE J.* **1972**, *18*, 122–127.

(S5) Calabrese, R. V.; Chang, T. P. K.; Dang, P. T. Drop breakup in turbulent stirred-tank contactors. Part I: Correlations for mean size and drop size distribution. *AIChE J.* **1986**, *32*, 657–666.

(S6) Wang, C.; Calabrese, R. V. Drop breakup in turbulent stirred-tank contactors. Part II: Relative influence of viscosity and interfacial tension. *AIChE J.* **1986**, *32*, 667–676.

(S7) Nishikawa, M.; Mori, F.; Fujieda, S. Average drop size in a liquid-liquid phase mixing vessel. *J. Chem. Eng. Japan* **1987**, *20*, 82–88.

(S8) Baldyga, J.; Bourne, J. Some consequences for turbulent mixing of fine-scale intermittency. *Chem. Eng. Sci.* **1992**, *47*, 3943–3948.

(S9) Das, P. K. Prediction of maximum stable diameter of viscous drops in a turbulent dispersion. *Chem. Eng. Tech.: Ind. Chemistry-Plant Equip.-Proc, Eng.-Biotech.* **1996**, *19*, 39–42.

(S10) Hall, S.; Pacek, A. W.; Kowalski, A. J.; Cooke, M.; Rothman, D. The effect of scale and interfacial tension on liquid–liquid dispersion in in-line Silverson rotor–stator mixers. *Chem. Eng. Res. Des.* **2013**, *91*, 2156–2168.

(S11) Liu, C.; Li, M.; Liang, C.; Wang, W. Measurement and analysis of bimodal drop size distribution in a rotor–stator homogenizer. *Chem. Eng. Sci.* **2013**, *102*, 622–631.
